# Urban-rural differences in the association between access to healthcare and health outcomes among older adults in China

**DOI:** 10.1186/s12877-017-0538-9

**Published:** 2017-07-19

**Authors:** Xufan Zhang, Matthew E. Dupre, Li Qiu, Wei Zhou, Yuan Zhao, Danan Gu

**Affiliations:** 1 Ginling College & International Center for Aging and Health, Nanjing Normal University, Nanjing, China; 2 Duke Clinical Research Institute & Department of Sociology, Duke University, Durham, NC USA; 3 Independent Researcher, New York, NY USA; 4 Ginling College, School of Geography Science & International Center for Aging and Health and Nanjing Normal University, Nanjing, China; 5 United Nations Population Division, Two UN Plaza, New York, NY DC2-1910 USA

**Keywords:** Healthcare, Medical care, Access to healthcare, Disability, Mortality, Older adults, Oldest-old, Rural, Rrban, China, CLHLS

## Abstract

**Background:**

Studies have shown that inadequate access to healthcare is associated with lower levels of health and well-being in older adults. Studies have also shown significant urban-rural differences in access to healthcare in developing countries such as China. However, there is limited evidence of whether the association between access to healthcare and health outcomes differs by urban-rural residence at older ages in China.

**Methods:**

Four waves of data (2005, 2008/2009, 2011/2012, and 2014) from the largest national longitudinal survey of adults aged 65 and older in mainland China (*n* = 26,604) were used for analysis. The association between inadequate access to healthcare (y/n) and multiple health outcomes were examined—including instrumental activities of daily living (IADL) disability, ADL disability, cognitive impairment, and all-cause mortality. A series of multivariate models were used to obtain robust estimates and to account for various covariates associated with access to healthcare and/or health outcomes. All models were stratified by urban-rural residence.

**Results:**

Inadequate access to healthcare was significantly higher among older adults in rural areas than in urban areas (9.1% vs. 5.4%; *p* < 0.01). Results from multivariate models showed that inadequate access to healthcare was associated with significantly higher odds of IADL disability in older adults living in urban areas (odds ratio [OR] = 1.58–1.79) and rural areas (OR = 1.95–2.30) relative to their counterparts with adequate access to healthcare. In terms of ADL disability, we found significant increases in the odds of disability among rural older adults (OR = 1.89–3.05) but not among urban older adults. Inadequate access to healthcare was also associated with substantially higher odds of cognitive impairment in older adults from rural areas (OR = 2.37–3.19) compared with those in rural areas with adequate access to healthcare; however, no significant differences in cognitive impairment were found among older adults in urban areas. Finally, we found that inadequate access to healthcare increased overall mortality risks in older adults by 33–37% in urban areas and 28–29% in rural areas. However, the increased risk of mortality in urban areas was not significant after taking into account health behaviors and baseline health status.

**Conclusions:**

Inadequate access to healthcare was significantly associated with higher rates of disability, cognitive impairment, and all-cause mortality among older adults in China. The associations between access to healthcare and health outcomes were generally stronger among older adults in rural areas than in urban areas. Our findings underscore the importance of providing adequate access to healthcare for older adults—particularly for those living in rural areas in developing countries such as China.

## Background

Healthcare is inarguably one of the most well-known and widely studied determinants of health [1–4]. Adequate access to healthcare is especially critical to older adults who require greater levels of treatment and care to manage disease and prolong survival [5]. Indeed, a number of studies have shown that adequate access to medical services significantly improves the health and longevity of older adults [4, 6–12]. Previous studies have also suggested that access to healthcare varies across different segments of the population—e.g., by age, sex, race, and urban-rural residence [13–15]. However, much of this research is based on Western nations and few studies have examined the impact of access to healthcare on health outcomes in developing nations such as China. Moreover, it is largely unknown whether access to healthcare has similar benefits in urban and rural areas in China where there are well-known disparities in socioeconomic status and other risks/resources [7, 16, 17].

Since 1950, China has implemented a dual-system of social welfare in urban and rural areas [7]. As a result, the healthcare system in rural areas is entirely different from the healthcare system in urban areas [14, 16–23]. In rural areas, the current healthcare insurance system is the New Cooperative Medical Scheme (NCMS), which was launched in the early 2000s after the old cooperative medical scheme collapsed following economic reforms in the late 1970s [24, 25]. In urban China, the current health insurance system, or urban medical schemes (UMS), mainly consists of the Urban Employer-sponsored Medical Scheme (UEMS, for urban employees) and the Urban Resident Medical Scheme (URMS, for urban residents without UEMS). The UEMS system was launched in 1999 and was mainly a modification of the free public medical scheme that existed in the planned economy. The URMS system is relatively new and implemented nationally in 2010 after a three-year pilot program [26–28].

Previous studies have demonstrated significant urban-rural differences in access to and utilization of healthcare in China. For example, research shows that older adults from rural areas in China are less likely to use inpatient services and more likely to rely on in-home medical care than their urban counterparts [18, 20]. Considering the unique systems of stratification and healthcare in China and the limited research on urban-rural differential in links between healthcare assess and health outcomes, the purpose of the current study is to investigate whether the association between access to healthcare and health outcomes varies by urban-rural residence among older Chinese. Using nationally representative longitudinal data from China, we examined whether and to what extent adequate access to healthcare was related to disability, cognitive impairment, and all-cause mortality among older adults residing in urban and rural areas. The implications of the findings are discussed in the context of rapidly aging societies such as China and the need for sustainable healthcare resources for older adults.

## Methods

### Sample

This study uses four waves of data from the Chinese Longitudinal Healthy Longevity Survey (CLHLS) of adults aged 65 and older from mainland China. The data for analysis were drawn from surveys conducted in 2005, 2008/2009 (2008 for short), 2011/2012 (2011 for short), and 2014. The first three waves of the CLHLS (1998, 2000, and 2002) were not included in the study because the ascertainment of the respondents’ access to healthcare was not consistent and data indicating healthcare insurance were not collected. The CLHLS was conducted in a randomly selected half of the counties/cities in 23 provinces throughout China. Eight other provinces were excluded from the sampling design to avoid known inaccuracies in age-reporting at very old ages in these locations [29]. The total population of the 23 provinces accounts for nearly 90% of the total population in China according to the 2010 census. The CLHLS was uniquely designed to provide a large replenished (age-sex-specific) oversampling of octogenarians (ages 80–89), nonagenarians (ages 90–99), and centenarians (ages 100+) to provide robust data to study aging and longevity in China. Respondent information was obtained through in-home interviews (approximately two hours in length) and used various other data sources—e.g., birth certificates, genealogical documents, household booklets, and/or the ages of children and siblings—when available to validate the accuracy of age reports. Each sampled individual provided a written informed consent to indicate his/her willingness to participate in the CLHLS. The informed consent was signed by the next-of-kin in the case when the respondent was not able to write. Overall response rates for each wave of data collection were approximately 98%. Further details of the sampling design, response rates, and overall data quality have been documented extensively elsewhere [29]. For the purposes of this study, the four waves of CLHLS data were pooled together to provide more robust estimates as recently shown [30]. The analytic sample includes 26,604 older adults (aged 65+) who were interviewed from 2005 to 2014—contributing 48,476 observations for analysis during this period.

### Measurement

#### Urban and rural definitions

The CLHLS classified participants according to their urban or rural residence at the time of the survey. Urban and rural areas in the CLHLS were defined by using definitions released by the National Bureau of Statistics of China (NBSC) in 1999 [31] for the 2005 wave and by the definition released by NBSC in 2006 [32] for the 2008 wave and beyond.

#### Adequate access to healthcare services

Healthcare includes a wide range of services, including preventative care, chronic disease management, emergency services, mental health services, dental care, and other community services that promote health over the lifespan [33]. In the CLHLS, healthcare was defined in the same context and included several questions on the respondents’ access to healthcare services. For this study, we used the question of whether respondents reported having adequate access to healthcare (medical care) services when needed (yes vs. no). To reduce missing data on this question, the CLHLS included responses from proxies (i.e., family members, etc.) to ascertain information on access to healthcare for participants who were too sick to provide such information.

#### Health outcomes

Health outcomes included measures for rates (i.e., prevalence) of disability in instrumental activities of daily living (IADLs) and ADLs, cognitive impairment, and all-cause mortality. Preliminary analyses also assessed measures for the incidence and recovery of limitations in IADLs, ADLs, and cognitive function. With one exception of the incidence of cognitive impairment, all other associations between access to healthcare and these measures were not significant; and thus not presented here (available upon request).

#### IADL disability, ADL disability, and cognitive impairment

Instrumental activities of daily living were measured based on eight self-reported activities: (a) visiting neighbors, (b) shopping, (c) cooking, (d) washing clothes, (e) walking one kilometer, (f) lifting 5 kg, (g) crouching and standing up three times, and (h) taking public transportation. Response categories for each item were: “able to do without help,” “need some help,” and “need full help.” The IADL items in the CLHLS were adopted from the Lawton scale [30]. We categorized respondents as IADL disabled (coded as 1) if they reported needing any help in performing any of the eight items (otherwise coded as 0).

Activities of daily living were measured based on six activities: (a) bathing, (b) dressing, (c) indoor transferring, (d) toileting, (e) eating, and (f) continence [30]. Using similar response categories as IADLs, we categorized respondents as ADL disabled (coded as 1) if they reported needing any help in performing any of the six items (otherwise coded as 0).

Cognitive function was measured using the Mini-mental Status Examination (MMSE) that includes six domains of cognition—i.e., orientation, reaction, calculation, short-term memory, naming, and language—with a total score of 30. Respondents were categorized as cognitively impaired if his/her MMSE score was below 24 [34]. Given the low level of educational attainment among most older adults in China, we assessed alternative criteria (e.g., score of 18) for those with no education to test the sensitivity of different cut-points for defining cognitive impairment. Results were consistent with those presented here (available upon request). As previously reported, the Chinese version of the MMSE used in the CLHLS was culturally translated from the standard version of the MMSE questionnaire [34]. The validity and reliability of the MMSE measure has been carefully tested in pilot surveys and verified in each wave of the CLHLS [29, 34].

#### Mortality

Survival status was defined as whether a respondent was dead or alive at the time of the 2014 survey. Exposure to mortality risk was ascertained by the number of days alive from the date of the first interview (baseline) to either a) the date at death for those who died before the 2014 survey or b) the date of the 2014 survey for those who were still alive during the study period. From 2005 to 2014, about 50% of individuals died (27% in the weighted data); and approximately 21% of respondents (28% weighted) were lost to follow-up and ultimately excluded because there was no information regarding the survival status or the length of exposure for these individuals. Previous studies have demonstrated that the quality of mortality data is high in the CLHLS [29, 35].

#### Covariates

The analyses include a variety of sociodemographic and behavioral covariates that have been previously linked to access to healthcare [1, 5, 36–39], IADL and ADL disabilities, cognitive impairment, and overall mortality [7, 40–42]. Sociodemographic covariates included age, sex, marital status (currently married vs. not married), coresidence with children (yes vs. no), years of schooling (0, 1–6, and 7+), primary lifetime occupation (white collar occupation vs. others), and economic independence (having a retirement wage/pension and/or own earnings vs. no). Next, two additional variables were included to assess whether participants were enrolled in the national health insurance programs in China. The variables included whether the respondent was enrolled in NCMS (yes vs. no) and whether the respondent was enrolled (yes vs. no) in one of the two UMS (i.e., UEMS and URMS) (some of very old urban cohorts may be still covered by public free medical program). Finally, health behaviors included whether the respondent was a smoker (never, quit, or currently smoking) at the time of survey and the frequency of regular engagement in leisure activities. The measure of leisure activities included six activities: (a) playing cards/mahjong, (b) watching TV, listening to the radio, or accessing the internet, (c) reading books or newspapers, (d) raising pets or domestic poultry, (e) gardening or doing housework, and (f) engaging in outdoor activities such as exercise or jogging. Each item was scored from 0 (no participation) to 4 (daily participation) and summed to capture the overall frequency of involvement in leisure activities. The summed scores were eventually classified into four groups: 0, 1–9, 10–14, and 15–24. All analyses also controlled for the CLHLS survey year (2005, 2008, 2011, and 2014) and included an indicator for proxy responses (yes vs. no) to the question of access to healthcare.

#### Analytical strategy

Multivariate logistic regression models were used to examine the association between inadequate access to healthcare and the prevalence of IADL disability, ADL disability, and cognitive impairment stratified by urban-rural residence. The stratification was justified by the distinction of health insurance programs between urban and rural areas and by statistical differences between urban and rural areas in the associations between access to healthcare and cognitive impairment (*p* < 0.001) and ADL disability (*p* < 0.1). Three nested models were used to examine how the different covariates contributed to the associations. First, Model I controlled for survey year, proxy responses to healthcare access, and included covariates for sociodemographic background. Preliminary analyses assessed separate models for demographics and socioeconomic status (SES); however, the results were largely consistent and the models were subsequently combined. Next, Model II included Model I covariates and further added measures for respondents’ enrollment statuses in the national health insurance programs. The final model (Model III) further included participants’ health behaviors.

Cox proportional hazard models were used to examine the association between inadequate access to healthcare and the risk of all-cause mortality. Based on our preliminary analyses, some of the variables violated the proportionality assumption. Further inclusion of interaction terms to account for the length of mortality exposure during the survey period did not alter the results; therefore, we present results without the added interaction parameters. Study participants who were lost to follow-up were excluded from the analyses. Similar results were obtained using an alternative strategy that included those lost to follow-up by imputing the length of their exposure and survival status. The regression-based imputation assumed that respondents lost to follow-up would have the same length of exposure and survival status if they had the same characteristics as those who were not lost to follow-up. As described above, a series of nested models were used to examine IADL disability, ADL disability, and cognitive impairment. An additional model (Model IV) was included to account for baseline health status.

All covariates in the multivariate models for the prevalence of disability and cognitive impairment were included as time-varying measures—with one exception of gender—and adjusted for the intrapersonal correlation due to multiple observations from respondents. For the mortality analyses, the covariates were fixed at baseline to estimate risk over the entire study period (i.e., 2005 to 2014). Supplementary analyses also used time-varying covariates and adjustments for intrapersonal correlation to estimate 3-year mortality in each survey interval between two adjacent waves and the results were comparable. Missing data among all covariates was generally low (less than 2%) and multiple imputation methods were used to minimize potential bias attributable to missing values. Alternative approaches were also assessed (e.g., mean or mode imputation, etc.) and the results were nearly identical. We also assessed possible multicollinearity among variables and found that all variance inflation factors were less than 3 [43]. All analytical models used weights to account for the sampling design of the CLHLS. All analyses were performed using Stata version 13.1.

## Results

Table 1 presents the weighted distributions of the total sample and by urban-rural residence. Although the reported access to healthcare was generally high among all CLHLS respondents, older adults in rural areas were nearly twice as likely as older adults in urban areas to report inadequate access to healthcare (9.1% vs. 5.4%; *p* < 0.01) during the period 2005–2014. Overall, older adults in rural areas had lower levels of SES, were more likely to smoke, spend less time on leisure activities, and had lower levels of ADL disability than older adults in urban areas. Nearly half of rural older adults (47.4%) were enrolled in NCMS and an additional 7.2% were enrolled in the MS insurance program. Among urban older adults, almost a quarter were enrolled in NCMS (24.9%) and more than a third (37.6%) were enrolled in one of three urban healthcare insurance programs.

**Table 1:** Weighted percentages of study variables by urban-rural residence among adults aged 65 and older in China, CLHLS, 2005–2014

	Total	Urban	Rural	*P* values
#, Total individuals (unweighted)	26,604	10,582	16,022	
Access to healthcare				
% inadequate access to healthcare	7.7	5.4	9.1	*p* < 0.010
Health outcomes				
% IADL disabled	32.0	32.3	31.7	*p* > 0.100
% ADL disabled	7.0	10.2	4.9	*P* < 0.001
% Cognitively impaired	13.3	11.9	13.6	*p* > 0.100
% Died in the period 2005–2014	26.8	26.1	27.2	*p* > 0.100
Sociodemographics				
Mean age (in years)	72.0	71.7	72.2	*p* < 0.100
% Men	49.2	48.3	49.8	*p* > 0.100
% Currently married	65.5	66.1	65.1	*p* > 0.100
% Coresidence with children	45.2	44.5	45.6	*p* > 0.100
% 0 years of schooling	42.0	30.5	49.4	*p* < 0.001
% 1–6 years of schooling	40.5	41.8	39.6	*p* < 0.001
% 7+ years of schooling	17.5	27.7	11.0	*p* < 0.001
% White collar occupation	12.1	22.2	5.6	*p* < 0.001
% Economic independence	51.8	63.7	44.2	*p* < 0.001
National health insurance enrollment				
% Enrolled in rural NCMS	38.6	24.9	47.4	*p* < 0.001
% Enrolled in urban MS	19.0	37.6	7.2	*p* < 0.001
Health behaviors				
% Never smoked	61.9	62.6	61.4	*p* < 0.05
% Quit smoking	13.0	15.6	11.4	*p* < 0.05
% Currently smoking	25.1	21.8	27.2	*p* < 0.05
Leisure activity index score 0	2.6	2.2	2.9	*p* < 0.001
Leisure activity index scores 1–9	26.9	22.4	29.7	*p* < 0.001
Leisure activity index scores 10–14	36.1	34.1	37.3	*p* < 0.001
Leisure activity index scores 15–24	34.4	41.3	30.0	*p* < 0.001
Survey measures				
% Wave 2005	50.8	55.6	47.7	*p* < 0.001
% Wave 2008	26.2	25.2	26.8	*p* < 0.001
% Wave 2011	10.9	3.6	15.6	*p* < 0.001
% Wave 2014	12.1	15.6	9.9	*p* < 0.001
% Proxy response for the adequate access question	4.2	3.8	4.5	*p* > 0.100

*IADL* instrumental activities of daily living, *ADL* activities of daily living, *NCMS* new cooperative medical scheme, *MS* medical scheme

Weighted percentages were based on the number of individuals. The percentages were similar to those based on the number of observations—with the exceptions of the distributions for survey wave and enrollment in medical scheme. The statistical test for urban-rural differences in weighted percentages was based on a Stata package “parmest” (see http://ideas.repec.org/p/boc/usug08/07.html)

Table 2 reports the odds ratios (ORs) for IADL disability associated with inadequate access to healthcare by urban and rural residence. Results show that inadequate access to healthcare was associated with 79% (OR = 1.79; *p* < 0.001) higher odds of IADL disability among older adults in urban areas and a 130% (OR = 2.30; *p* < 0.001) higher odds among older adults in rural areas when taking into account sociodemographic factors in Model I. The ORs remained largely unchanged when further taking into account enrollment in the national health insurance programs (Model II). We also found that there was no association between enrollment in urban medical schemes and the prevalence of IADL disability. Further adjustment for health behaviors in Model III shows that the ORs were partially attenuated for older adults in urban (OR = 1.58; *p* < 0.05) and rural (OR = 1.95; *p* < 0.001) areas.

**Table 2:** Odds ratios of IADL disability for inadequate access to healthcare by urban-rural residence among adults aged 65 and older in China, CLHLS 2005–2014

	Urban			Rural		
	Model I	Model II	Model III	Model I	Model II	Model III
Inadequate access to healthcare (no)	1.78***	1.80***	1.54*	2.31***	2.25***	1.99***
Sociodemographics						
Age	1.12***	1.12***	1.11***	1.12***	1.12***	1.11***
Men (women)	0.71**	0.73*	0.65*	0.57***	0.57***	0.51***
Currently married (no)	0.67***	0.68***	0.73**	0.85**	0.86*	0.94
Coresidence with children (no)	0.91	0.91	0.89	1.00	0.99	1.03
1–6 years of schooling (0)	0.86	0.84	0.91	0.85^+^	0.85*	0.93
7+ years of schooling (0)	0.78	0.72^+^	0.87	0.75*	0.72*	0.84
White-collar job (no)	1.02	0.98	1.05	1.51***	1.43**	1.36*
Economic independence (no)	0.71**	0.63***	0.72*	0.48***	0.47***	0.52***
National health insurance enrollment						
Enrolled in rural NCMS (no)		0.71*	0.80		0.79*	0.90
Enrolled in urban MS (no)		1.04	1.24		1.09	1.15
Health behaviors						
Quit smoking (never)			1.35*			1.38***
Currently smoking (never)			0.80			0.81*
Leisure activity scores 1–9 (0)			0.31***			0.22***
Leisure activity scores 10–14 (0)			0.12***			0.11***
Leisure activity scores 15^+^ (0)			0.07***			0.09***
N (observations)	21,038	21,038	21,038	27,154	27,154	27,154
Wald Chi square	852.5***	949.6***	1074.3***	1313.7***	1459.5***	1683.9***

*IADL* instrumental activities of daily living, *NCMS* new cooperative medical scheme, *MS* medical scheme

Estimated odds ratios were weighted and adjusted for intrapersonal correlation. The total analytic sample was 26,604 individuals (*n* = 48,476 observations). All models also controlled for survey year and proxy responses to the question of adequate access to healthcare

^+^ *p* < 0.1, *p < 0.05, **p < 0.01, ***p < 0.001

Table 3 presents the ORs for ADL disability associated with inadequate access to healthcare by urban and rural residence. Results show that inadequate access to healthcare among rural older adults was associated with substantially higher rates of ADL disability after adjusting for sociodemographic factors (OR = 3.05; *p* < 0.001), enrollment in national health insurance (OR = 2.76; *p* < 0.001), and health behaviors (OR = 1.89; *p* < 0.001). In contrast, we found no significant differences in ADL disability associated with access to healthcare among older adults in urban areas. The results for cognitive impairment in Table 4 were largely similar with significantly higher odds in Model I (OR = 3.19; *p* < 0.001), Model II (OR = 2.94; *p* < 0.001), and Model III (OR = 2.37; *p* < 0.001) among rural older adults with inadequate access to healthcare compared to their counterparts with adequate access to healthcare. No significant differences in cognitive impairment associated with access to healthcare in urban areas were found.

**Table 3:** Odds ratios of ADL disability for inadequate access to healthcare by urban-rural residence among adults aged 65 and older in China, CLHLS 2005–2014

	Urban			Rural		
	Model I	Model II	Model III	Model I	Model II	Model III
Inadequate access to healthcare (no)	1.78+	1.71^+^	1.27	3.06***	2.77***	1.82***
Sociodemographics						
Age	1.05***	1.05***	1.02	1.06***	1.06***	1.04***
Men (women)	1.19	1.27	1.09	0.93	0.95	0.80^+^
Currently married (no)	0.70+	0.72^+^	0.85	0.85	0.90	1.06
Coresidence with children (no)	0.82	0.83	0.77^+^	1.06	1.09	1.14
1–6 years of schooling (0)	1.47*	1.40*	1.58*	0.95	0.93	1.09
7+ years of schooling (0)	1.20	1.05	1.29	1.12	1.05	1.33
White-collar job (no)	1.05	0.97	1.06	2.06**	1.82*	1.60*
Economic independence (no)	0.90	0.78	0.93	0.56***	0.53***	0.65**
National health insurance enrollment						
Enrolled in rural NCMS (no)		0.44**	0.50**		0.52***	0.72*
Enrolled in urban MS (no)		0.75	0.91		0.93	1.13
Health behaviors						
Quit smoking (never)			1.51*			1.45**
Currently smoking (never)			0.91			0.84
Leisure activity scores 1–9 (0)			0.24***			0.13***
Leisure activity scores 10–14 (0)			0.06***			0.05***
Leisure activity scores 15^+^ (0)			0.04***			0.05***
N (observations)	21,038	21,038	21,038	27,154	27,154	27,154
Wald Chi square	607.4***	631.3***	992.2***	907.9***	925.5***	1281.7***

*ADL* activities of daily living, *NCMS* new cooperative medical scheme, *MS* medical scheme

Estimated odds ratios were weighted and adjusted for intrapersonal correlation. The total analytic sample was 26,604 individuals (*n* = 48,476 observations). All models also controlled for survey year and proxy responses to the question of adequate access to healthcare

^+^ *p* < 0.1, *p < 0.05, **p < 0.01, ***p < 0.001

**Table 4:** Odds ratios of cognitive impairment for inadequate access to healthcare by urban-rural residence among adults aged 65 and older in China, CLHLS 2005–2014

	Urban			Rural		
	Model I	Model II	Model III	Model I	Model II	Model III
Inadequate access to healthcare (no)	1.30^+^	1.25	0.92	3.27***	3.01***	2.45***
Sociodemographics						
Age	1.07***	1.07***	1.04***	1.09***	1.09***	1.08***
Men (women)	0.85	0.87	0.86	0.81***	0.81**	0.74***
Currently married (no)	0.54***	0.55**	0.60**	0.75***	0.77**	0.85*
Coresidence with children (no)	0.70**	0.71**	0.66***	0.88+	0.89^+^	0.91
1–6 years of schooling (0)	0.68***	0.66**	0.70***	0.56***	0.56***	0.60***
7+ years of schooling (0)	0.41***	0.39***	0.45**	0.41***	0.40***	0.43***
White-collar job (no)	0.95	0.89	0.93	1.17	1.11	1.01
Economic independence (no)	0.54***	0.49***	0.58***	0.56***	0.55***	0.65***
National health insurance enrollment						
Enrolled in rural NCMS (no)		0.58**	0.66*		0.60***	0.73***
Enrolled in urban MS (no)		0.86	0.99		0.73*	0.77^+^
Health behaviors						
Quit smoking (never)			1.03			112
Currently smoking (never)			0.72+			0.93
Leisure activity scores 1–9 (0)			0.25***			0.24***
Leisure activity scores 10–14 (0)			0.09***			0.15***
Leisure activity scores 15^+^ (0)			0.05***			0.10***
N (observations)	21,038	21,038	21,038	27,154	27,154	27,154
Wald Chi square	1045.6***	1037.0***	1406.8***	1625.7***	1659.1***	1755.5***

*NCMS* new cooperative medical scheme, *MS* medical scheme

Estimated odds ratios were weighted and adjusted for intrapersonal correlation. The total analytic sample was 26,604 individuals (*n* = 48,476 observations). All models also controlled for survey year and proxy responses to the question of adequate access to healthcare

^+^ *p* < 0.1, *p < 0.05, **p < 0.01, ***p < 0.001

Table 5 reports the hazard ratios (HRs) for the association between inadequate access to healthcare and mortality by urban-rural residence. When controlling for sociodemographics (Model I), we found that inadequate access to healthcare was associated with 30% (*p* < 0.001) higher risks of mortality among rural older adults and 36% (*p* < 0.001) higher risks for urban older adults. Further accounting for enrollment in national health insurance programs (Model II) only slightly attenuated the association between access to health care and mortality in both urban and rural areas. Although we found that enrollment in NCMS was associated with significant reductions in mortality, NCMS enrollment had little impact on the association between access to healthcare and mortality in urban and rural older adults. Enrollment in urban medical schemes almost had no association with mortality. However, after taking into account health behaviors (Model III) and health status (Model IV), inadequate access to healthcare was not significantly associated with mortality among older adults living in urban areas. In contrast, inadequate access to healthcare increased risks for mortality by 23% (*p* < 0.01) and 16% (*p* < 0.05) among those in rural areas.

**Table 5:** Relative hazard ratios of mortality for inadequate access to healthcare by urban-rural residence among adults aged 65 and older in China, CLHLS 2005–2014

	Urban				Rural			
	Model I	Model II	Model III	Model IV	Model I	Model II	Model III	Model IV
Inadequate access to healthcare	1.37*	1.33*	1.18	1.15	1.29***	1.28***	1.24**	1.17*
Sociodemographics								
Age	1.09***	1.09***	1.08***	1.07***	1.10***	1.10****	1.09***	1.08***
Men (women)	1.53***	1.56***	1.28**	1.39***	1.57***	1.57***	1.40***	1.45***
Currently married (no)	0.98	0.99	0.98	0.98	0.96	0.97	0.96	0.98
Coresidence with children (no)	1.01	1.01	0.98	0.97	1.05	1.05	1.04	1.03
1–6 years of schooling (0)	0.82*	0.81*	0.88	0.89	0.99	0.99	1.02	1.05
7+ years of schooling (0)	0.73*	0.71**	0.83	0.84	0.91	0.90	0.94	0.97
White-collar job (no)	1.14	1.13	1.12	1.12	1.18^+^	1.17^+^	1.15	1.12
Economic independence (no)	0.78**	0.76**	0.81*	0.82*	0.75***	0.75***	0.80**	0.84**
National health insurance enrollment								
Enrolled in rural NCMS (no)		0.70***	0.74**	0.73**		0.84*	0.86*	0.86*
Enrolled in urban MS (no)		0.96	1.09	1.07		1.01	1.01	1.03
Health behaviors								
Quit smoking (never)			1.37**	1.26*			1.40***	1.36***
Currently smoking (never)			1.36***	1.38***			1.07	1.10
Leisure activity scores 1–9 (0)			0.52***	0.64***			0.52***	0.66***
Leisure activity scores 10–14 (0)			0.35***	0.48***			0.40***	0.53***
Leisure activity scores 15^+^ (0)			0.27***	0.38***			0.39***	0.54***
Baseline health								
IADL disabled (no)				1.40***				1.43***
ADL disabled (no)				1.46***				1.58***
Cognitively impaired (no)				1.18*				1.15*
N (individuals)	7588	7588	7588	7588	12,926	12,926	12,926	12,926
Wald Chi square	631.3***	664.0***	866.7***	908.2***	1284.7***	1303.6***	1521.9***	1573.8***

*IADL* instrumental activities of daily living, *ADL* activities of daily living, *NCMS* new cooperative medical scheme, *MS* medical scheme

The total analytic sample was 20,514 individuals (excluding those lost to follow-up). All models also controlled for survey year and proxy responses to the question of adequate access to healthcare

^+^ *p* < 0.1, *p < 0.05, **p < 0.01, ***p < 0.001

## Discussion

This study examined the association between access to healthcare and multiple health outcomes in a large nationally representative longitudinal dataset of adults aged 65 and older in China. To our knowledge, this study was the first to investigate whether and to what extent inadequate access to healthcare is associated with physical disability, cognitive impairment, and mortality in urban and rural older adults in a developing country. We found that older adults with inadequate access to healthcare had significantly higher rates of IADL disability, ADL disability, cognitive impairment, and all-cause mortality compared with older adults with adequate access to care. The associations were stronger among older adults in rural areas than in urban areas; and the associations persisted despite adjusting for multiple demographic, socioeconomic, behavioral, and health-related factors.

Adequate access to healthcare is beneficial to health by facilitating timely and quality medical care to screen for and treat diseases in early stages, postponing declines in physiological function with illness, restoring immune function, and ultimately prolonging survival. We showed that access to healthcare has significant impact on multiple health outcomes in older adults and that inadequate access to healthcare is especially detrimental to the health of older adults residing in rural locations. Our findings build on previous studies that have shown associations between increases in health insurance coverage and health outcomes [9, 10, 44, 45] and studies that have shown associations between access to healthcare or health insurance and mortality among the elderly population [7], the elderly in urban areas [44], and among patients with specific diseases [12].

In rural China today, the majority of the older adults can access to healthcare and can receive medical treatment for their health problems. This is because the Chinese healthcare system has greatly improved in recent years, with coverage reaching more than 95% of the population as of 2015 in both urban and rural areas [26]. In most rural areas, local/village doctors and nurses are widely available and physician visits are relatively inexpensive and prescription medications are generally accessible [20]. Nevertheless, there are rural residents who do not have adequate access to healthcare and are often the most vulnerable, frail, and poorest older adults who are unable to receive preventative care and/or timely treatment(s). Although the healthcare system has been greatly improved in rural areas [46], the health insurance program in rural China is not as good (or beneficial) as programs in urban areas. In rural areas, financial barriers are often considered the underlying reasons for inadequate access to healthcare [16]—and largely attributable to the lack of pensions, lower income, higher out-of-pocket cost, and greater co-payments compared with urban areas [47]. Consequently, rural older adults may face disproportionately greater difficulties in getting adequate medical services when needed relative to their urban counterparts, which lead to a high heterogeneity in rural areas between those who are able to adequately access to healthcare and those who are not able to do so.

According to the 2005–2014 waves of the CLHLS, approximately 60% of rural older adults who reported inadequate access to healthcare expressed financial constraint as the main barrier to accessing medical services. Among urban older adults, only 45% expressed financial constraints to accessing care. Older adults in rural areas also face obstacles to care from family members (i.e., sons and daughters) who have increased migrated to urban areas for education and work opportunities [48]. In contrast, older adults in urban areas are more likely to use hospital services and generally benefit from higher quality of care, better medical facilities, and more advanced treatment options. Notwithstanding, these advantages in urban settings have been hampered by a rapidly growing elderly population and the over-utilization of medical services—which has led to longer waiting times, delayed treatments, and ultimately less timely and effective treatments [23, 26]. As a result, the health disparity between older adults with and without adequate access to healthcare in urban areas is less discernible than in rural areas.

A unique feature of the present study is the measure of (inadequate) access to healthcare. This approach is different from most previous studies that focused exclusively on whether having health insurance is associated with a given health outcome [9, 10, 41, 49–53]. Although informative, researchers have argued that comparisons of health outcomes by basic categorizations of insured or uninsured make it difficult to determine the nature of the association—i.e., whether having insurance impacts health or whether health status impacts having insurance [41]. As such, this issue may explain, in part, why many previous studies in various settings find no significant association between having health insurance and mortality [49, 50, 54, 55]. The measure of inadequate access to healthcare used in the current study avoided such an issue of endogeneity.

One strength of our study was the simultaneous examination of multiple health outcomes associated with access to healthcare. We are aware of only one recent study that examined the association between enrollment in a health insurance program in China and several quality-of-life indicators [10]. We built on this research by demonstrating robust associations between access to healthcare and the prevalence of physical disability, cognitive impairment, and all-cause mortality. Moreover, we demonstrated that the associations were not uniform among urban and rural older adults after adjustment for several demographic, socioeconomic, behavioral, and health-related factors. Another strength of the study was the use of a large-scale nationally representative sample of older adults from a developing country that were examined over almost a 10-year period (2005–2014).

Our findings also have potentially important implications for improving access to healthcare in China. Given the high coverage rate of the three pillars of healthcare coverage in China (NCMS, UEMS, and URMS), these health insurance schemes have made most older adults enable to afford basic healthcare services when needed [24, 26]. However, there have been dramatic increases in the need and utilization of healthcare services due to a number of factors, including: (i) a rapidly aging population, (ii) a shortage of healthcare professionals, (iii) reduced familial caregiving due to smaller family size, (iv) sky-rocketing costs of medications and medical services, and (v) complicated reimbursement procedures for utilization. In addition, rural older adults face disproportionate challenges relative to their urban counterparts in obtaining timely and adequate medication due to high co-payments in NCMS. Based on the findings from this study, inadequate access to healthcare can be especially detrimental to health in rural areas. It is perhaps that increasing the benefits under NCMS or the unification of urban-rural healthcare programs could be viable strategies toward reducing health disparities faced by older adults in rural areas of China.

Despite the strengths of this study, we acknowledge several limitations. First, the measure of (in)adequate access to healthcare in this study is based on the respondents’ self-reported perceptions of access. We recognize that such self-assessments may not reflect actual access (or use) of healthcare and may be influenced by differences in background (e.g., age, sex, education), resources (e.g., income), and/or health status. Furthermore, respondents’ perceptions may be based on past experiences with healthcare—particularly among those without disability or impairment at the time of the survey. However, research suggests that self-perceived access to healthcare is highly aligned with actual use of healthcare services; including overall utilization, use of mental health services, routine checkups, and emergency care [4, 56, 57]. Therefore, we followed the existing literature by defining access to healthcare as a measure of obtaining needed healthcare services—rather than actual utilization—as an indicator of access to positive health outcomes [58]. Nevertheless, we encourage additional research with comparable (or alternative measures) of adequate access to healthcare to further validate and build on the current findings. A second limitation is that we did not consider the severity of each health outcome. Therefore, we acknowledge that individual needs for healthcare may differ according to the severity of the condition. Third, as the length of stay in the current residential status was not available in the CLHLS, we were not able to model the urban-rural residential change on the effect of inadequate access to healthcare. Research that includes such information is preferable in studying urban and rural disparities in access to healthcare. Finally, we could not control for potentially important factors related to healthcare use. For example, we lacked important contextual measures such as the actual availability of healthcare services, barriers to access (e.g., transportation, distance, terrain), and the quality of healthcare that is provided [1, 59, 60]. Therefore, more research is needed to further investigate these factors and others to better understand the association between access to healthcare and health outcomes in urban and rural settings in developing countries such as China.

## Conclusions

Inadequate access to healthcare is significantly associated with higher rates of disability, cognitive impairment, and all-cause mortality among older adults in China. The associations between access to healthcare and health outcomes were generally stronger among older adults in rural areas than in urban areas. Our findings underscore the importance of providing adequate access to healthcare for older adults—particularly for those living in rural areas in developing countries. In the context of near-universal healthcare coverage in China, the results identify opportunities to further improve access in rural areas to improve health outcomes in disadvantaged older adults.

## Abbreviations

ADL: Activities of daily living
CLHLS: Chinese Longitudinal Healthy Longevity Survey
HR: Hazard ratio
IADL: Instrumental activities of daily living
MMSE: Mini-mental Status Examination
NCMS: New Cooperative Medical Scheme
OR: Odds ratio
SES: Socioeconomic status
UEMS: Urban Employer-sponsored Medical Scheme
UMS: Urban medical scheme
URMS: Urban Resident Medical Scheme
